# Associations between the Expression of Epigenetically Regulated Genes and the Expression of DNMTs and MBDs in Systemic Lupus Erythematosus

**DOI:** 10.1371/journal.pone.0045897

**Published:** 2012-09-21

**Authors:** Eva Balada, Jesús Castro-Marrero, Lledó Felip, Josep Ordi-Ros, Miquel Vilardell-Tarrés

**Affiliations:** Research Unit in Systemic Autoimmune Diseases, Vall d'Hebron Research Institute, Hospital Vall d'Hebron, Universitat Autónoma de Barcelona, Barcelona, Spain; University of Patras Medical School, Greece

## Abstract

**Objectives:**

We determined the expression of ITGAL, PRF1, KIR2DL4, CD70, and CD40LG in patients with SLE and performed correlations with the global DNA methylation status and the levels of three DNA methylation enzymes and two methyl CpG-binding domain (MBD) proteins.

**Patients and Methods:**

CD4^+^ T cells were isolated from 35 SLE patients and 30 healthy controls. DNA deoxymethylcytosine content was measured by an enzyme-linked immunosorbent assay (ELISA). Transcript levels of ITGAL, PRF1, KIR2DL4, CD70, CD40LG, DNMT1, DNMT3A, DNMT3B, MBD2, and MBD4 were quantified by real-time reverse-transcription polymerase chain reaction (RT-PCR).

**Results:**

SLE patients had significantly elevated transcript levels of ITGAL (18.61±22.17 vs. 7.33±9.17, p = 0.042), PRF1 (21.67±26.34 vs. 10.67±11.65, p = 0.039), and CD70 (1.45±1.63 vs. 0.67±0.28, p = 0.011). A positive correlation was observed between transcript levels of CD40LG and ITGAL (r = 0.477, p = 0.004) as well as between CD40LG and PRF1 (r = 0.557, p = 0.001). Transcript levels of KIR2DL4 were higher than controls' but it did not reach statistical significance (1.36±3.52 vs. 0.22±0.79, p = 0.560). A tight relationship with global DNA hypomethylation as well as with the expression of most of the DNA methylation-related genes was observed, especially for ITGAL, PRF1, and CD40LG.

**Conclusions:**

ITGAL, PRF1, and CD70 are overexpressed in SLE CD4^+^ T cells. The tight association of CD40LG with ITGAL and PRF1 leads us to infer that it probably contributes to the pathogenesis of the disease. The apparent simultaneous regulation between their expression and the global DNA hypomethylation as well as with the transcription of many DNA methylation-related enzymes, reinforces the idea that epigenetic mechanisms are responsible for the deregulation of ITGAL, PRF1, and CD40LG.

## Introduction

Epigenetics is currently considered an exciting and dynamic field of biological research that can help to discern the mechanisms of some complex diseases. One of such diseases is systemic lupus erythematosus (SLE). SLE is an autoimmune disorder characterized by T lymphocyte autoreactivity and the presence of autoantibodies directed against nuclear antigens. These autoantibodies lead to immune complex deposition and widespread tissue damage. Although the cause is unknown, both genetic and environmental factors seem to play a role on its etiopathology. Hence, it is plausible to believe that the interaction established between the genome under the influence of external factors (i.e. epigenetics) may have the key for understanding the disease.

DNA methylation is one of the major epigenetic mechanisms that contribute to the epigenome of a cell. In mammals it mainly occurs at cytosine residues found within CpG dinucleotides and it leads to the formation of 5-methylcytosine (5-^m^C). The enzymes which methylate DNA are known as DNA cytosine-5-methyltransferases (DNMTs). Well-known DNA methylating enzymes are DNMT1, DNMT3A, and DNMT3B. On the other hand, there are other enzymes that seem to actively demethylate DNA. In the past decade, just two methyl CpG-binding domain (MBD) proteins, MBD2 and MBD4, were considered to exert a catalytic action that could lead to DNA demethylation in mammal cells [1], [2]. Nevertheless, some other molecules have recently been suggested as important participants involved in active DNA demethylation [3].

T-cell DNA demethylation plays an important role in the pathogenesis of SLE. Thus, some medications that cause drug-induced lupus (procainamide, hydralazine) as well as ultraviolet light (which triggers lupus flares), can inhibit DNA methylation in cloned T cell lines and can induce self-reactivity [4]. Methylation levels in thymus and lymphatic nodules of a murine model of lupus (MRL/lpr) have been shown to be lower than those found in the MRL/+ strain [5]. CD4^+^ T cells of mice treated with methylation inhibitors (5-azacytidine (5-aza-C) or procainamide) and transferred to syngenic mice induce a glomerulonephritis mediated by immunocomplexes, as well as IgG anti-DNA and anti-histone antibodies [6]. Thus, it seems that methylation inhibition is sufficient to cause a lupus-like illness. As a matter of fact, several studies have proved that DNA extracted from T cells of SLE patients is globally hypomethylated when compared to DNA from normal T cells [7]–[9]. The causes of this hypomethylation are beginning to be elucidated. Some authors believe that it may be due to a defective extracellular receptor-associated kinase (ERK) pathway. Thus, it seems that signaling via this pathway is decreased in CD4^+^ T cells from SLE patients, causing a decreased DNMT1 expression [10], [11]. Recent works have proved that the overexpression of some microRNA (miRNA) in SLE CD4^+^ T cells also contributes to DNA demethylation by targeting DNMT1 [12], [13]. Other authors, though, believe that the DNA hypomethylation observed in SLE may be caused by an overexpression of proposed DNA demethylating enzymes, such as MBD2 and MBD4 [8], [14], [15]. Recent studies have demonstrated that an enzyme which also seems to be involved in DNA demethylation, the growth arrest and DNA-damage-inducible protein alpha (GADD45α), is overexpressed in CD4^+^ T cells from SLE patients [16].

The majority of CpG sites (70–80%) in human DNA are methylated and many of the non-methylated sites are found in the so-called CpG islands, which are normally on functioning promoters. A strong and direct correlation between promoter's DNA methylation and genetic inactivity is usually found. Therefore, the consequence of the defective capacity to methylate the DNA found in SLE T cells is that several methylation-sensitive promoting genes can be overexpressed. One of such genes is ITGAL, which encodes the integrin alpha L chain, one structural part of the lymphocyte function-associated antigen-1 (LFA-1). LFA-1 plays a central role in leukocyte intercellular adhesion and it also functions in lymphocyte costimulatory signaling. LFA-1 overexpression causes T cell autoreactivity *in vitro* and a lupus-like disease *in vivo* [17], [18]. Interestingly, DNA hypomethylation in SLE affects sequences flanking the ITGAL promoter [19] and an overexpression occurs on T cells from active SLE patients [20]. Perforin 1 (PRF1) has also been identified as a gene whose transcription is increased in CD4^+^ T cells from active SLE patients and it seems to be due to demethylation of a conserved region located between the promoter and upstream enhancer [21]. This aberrant overexpression of PRF1 in CD4^+^ T cells may contribute to the killing of autologous monocytes/macrophages by T cells observed in SLE [20]. Killer cell Ig-like receptor (KIR) gene family is also methylation-sensitive in human T cells. Less methylation of the KIR2DL4 promoter, for example, has been detected in SLE patients [22] Although KIRs are preferentially expressed on NK cells, T cells from lupus patients have higher levels of KIR genes and their expression seems to be proportional to disease activity [22].

The expression of some B-cell costimulatory molecules on CD4^+^ T cells is also affected in SLE. This is the case of CD70 and CD40LG. CD70 gene encodes a member of the tumor necrosis factor (TNF) which regulates B-cell activation and immunoglobulin synthesis. The overexpression of CD70 found on T cells from patients with active SLE has been shown to overstimulate the production of IgG [23]. CD40LG is located in X-chromosome and it has B-cell costimulatory functions resembling those of CD70. Women with SLE have been shown to overexpress CD40LG on CD4^+^ T cells [24] which, in turn, also overstimulate B cells to produce IgG [25].

The study of the expression of ITGAL, PRF1, KIR2DL4, CD70, and CD40LG genes in SLE patients was first prompted by the observation that these genes were overexpressed when DNA hypomethylation was induced *in vitro* with DNA methylation inhibitors. Several authors are currently performing genome-wide DNA methylation studies directly in CD4^+^ T cells or white blood cells from SLE patients. These works have been conducted in the general SLE population [26] and in monozygotic twins discordant for the disease [27]. This way, the promoters of other genes have also been proved to be hypomethylated.

In the present work, we focused our attention on ITGAL, PRF1, KIR2DL4, CD70, and CD40LG (i.e., the five most extensively studied SLE epigenetically-regulated genes) and we investigated for the first time their simultaneous expression. Furthermore, we performed correlation analyses of the transcript expression of these genes with the global DNA methylation status and the levels of several DNA methylation enzymes.

## Patients and Methods

### Patients

Data were collected from 35 Caucasian individuals (7 men and 28 women; mean age: 34.54 yrs, range: 20–64 yrs) who suffered from SLE. An ethnically matched random healthy control population (blood donors) was also included in the study (*n* = 30, 16 men and 14 women; mean age: 36.93 yrs, range: 21–66 yrs). Subjects' written consent was obtained according to the Declaration of Helsinki, [28] and the design of the work conformed to standards currently applied in Spain. All the SLE patients fulfilled at least four of the American College of Rheumatology criteria [29].

### Methods

#### Isolation of peripheral blood mononuclear cells (PBMCs) and CD4^+^ T cells

A total of 20 mL of ethylenediaminetetraacetic acid (EDTA)-K_3_-preserved venous peripheral blood were withdrawn from both patients and controls. Peripheral blood mononuclear cells (PBMCs) were obtained by Hystopaque-1077 (Sigma, Madrid, SPAIN) density gradient centrifugation. CD4^+^ T cells were isolated by negative selection with the CD4^+^ T Cell Isolation Kit II (Miltenyi Biotec, Bergisch Gladbach, Germany) using a cocktail of biotin-conjugated monoclonal antibodies against CD8, CD14, CD16, CD19, CD36, CD56, CD123, TCRγ/δ and glycophorin A, and magnetic microbeads conjugated to a monoclonal anti-biotin antibody. Thus, following the instructions provided by the manufacturer, magnetically labelled non-CD4^+^ T cells were depleted by retaining them on a MACS LS Column in the magnetic field of a MACS Separator (Miltenyi), while the unlabeled T helper cells passed trough the column and were collected. The purity of the enriched CD4^+^ T cells was evaluated by flow cytometry in a FACSCalibur flow cytometer (Beckton & Dickinson, Mountain View, CA, USA) after incubating an aliquot of the cell fractions (5×10^4^ cells) with 2 μL of an anti-CD4-FITC antibody (Miltenyi) for 10 min at 4°C. Purity of CD4^+^ T cells was generally higher than 90%.

#### Genomic DNA extraction and measurement of DNA deoxymethylcytosine (d^m^C) content by enzyme-linked immunosorbent assay (ELISA)

Cell DNA extraction was carried out with the QIAamp DNA Blood Midi kit (Qiagen, Hilden, Germany). DNA concentration and 260/280 absorbance ratios were calculated with a NanoDrop ND-1000 spectrophotometer (NanoDrop Technologies, Montchanin, DE, USA). DNA d^m^C content was measured by means of an ELISA developed in our laboratory as previously described [8].

#### RNA isolation and Real-Time quantitative-polymerase chain reaction (RT-PCR)

Total RNA from CD4^+^ T cells was isolated using the Ultraspec RNA isolation system (Biotecx Laboratories, Inc., Huston, TX, USA). Reverse transcription (RT) was carried out with the QuantiTect Reverse Transcription kit (Qiagen) according to the manufacturer's instructions. Two microliters of cDNA were taken for performing the PCR with the QuantiTect Multiplex PCR kit (Qiagen) in a total volume of 50 μL, which included: 25 μL of 2xQuantiTect Multiplex PCR master mix, 0.4 μM of each forward primer, 0.4 μM of each reverse primer, 0.2 μM of each probe, and 0.5 units of Uracyl-N-glycosilase (UNG) (Sigma, Madrid, Spain). Primers, probes, and thermocycling parameters to amplify the reference gene (β-actin) along DNMT1, DNMT3A, DNMT3B, MBD2, and MBD4 are described elsewhere [8], [30].

ITGAL, PRF1, KIR2DL4, CD70, and CD40LG, were quantified by using Taqman Gene expression assays from Applied Biosystems (Cheshire, United Kingdom). Primers (Forward: 5′-TCACCCACACTGTGCCCATCTACGA-3′, Reverse: 5′-CAGCGGAACCGCTCATTGCCAATGG-3′) and probe (Yakima Yellow-5′-ATGCCCTCCCCCATGCCATCCTGCGT-3′-BHQ1) sequences of the reference gene (β-actin) were from Eurofins MWG Synthesis GmbH (Ebersberg, Germany). Reactions for determining the expression of the gene of interest and β-actin were carried out as duplex PCRs (i.e., amplifying both genes in the same well) and all were run in duplicate in MicroAmp optical 96-well plates sealed with optical adhesive covers (Applied Biosystems) on an ABI PRISM 7000 Sequence Detection System. The amplification conditions comprised: 2 min at 50°C for UNG activation, 15 min at 95°C for HotStarTaq DNA polymerase activation, and 50 cycles consisting of 1 min at 94°C for denaturation and 1 min at 60°C for annealing/extension. Negative controls (in which water instead of cDNA was added) were also run in each plate. Furthermore, contamination of the RNA samples by genomic DNA was excluded by an analysis without prior cDNA conversion, i.e., excluding reverse transcriptase from the RT reaction. Each assay included a standard curve for both genes. The standard curve was constructed with serial dilutions of reverse transcription products corresponding to different concentrations of total RNA from a reference cell line (Jurkat). Since KIR2DL4 is known to be expressed in natural killer (NK) cells but not in Jurkat cells, [31], [32] we used the non-CD4^+^ T cell fraction (after flushing it out from the column with a plunger) from a healthy blood donor to obtain the RNA and construct the standard curve. Unknown sample expression levels were then determined from the standard curves and reported in equivalent quantity of total RNA from the reference cell line (or the non-CD4^+^T cell fraction). Normalization of RNA amounts was performed using β-actin expression analysed with the same procedure. Finally, expression ratios between the gene of interest and β-actin were calculated.

#### Statistical analysis

Either the Mann-Whitney U test or the independent samples t-test for equality of means (along with the Levene's test for equality of variances) were used to compare values. Spearman's rank correlation was used to examine the relationship between two continuous variables. *p* values less than 0.05 were considered significant. All analyses were performed with the Prism GraphPad 5.0 software.

## Results

### Transcript levels of ITGAL, PRF1, KIR2DL4, CD70, and CD40LG in CD4^+^ lupus T cells

We compared mRNA levels of ITGAL, PRF1, KIR2DL4, CD70, and CD40LG in CD4^+^ T cells from SLE patients and healthy controls. SLE patients had significantly elevated transcript levels of ITGAL (18.61±22.17 vs. 7.33±9.17, p = 0.042), PRF1 (21.67±26.34 vs. 10.67±11.65, p = 0.039), and CD70 (1.45±1.63 vs. 0.67±0.28, p = 0.011). Although KIR2DL4 transcript levels were also higher in SLE patients, it did not reach statistical significance (1.36±3.52 vs. 0.22±0.79, p = 0.560). Of note, 19 patients and 16 healthy controls did not express KIR2DL4 in their CD4^+^ T cells at all, and the values from 6 patients and 6 controls were not considered for the study since their KIR2DL4 mRNA levels were too low to obtain similar and reliable duplicate values. Statistically significant differences were not found for CD40LG either (18.69±13.01 vs. 18.75±10.99, p = 0.481).

**Figure 1:**
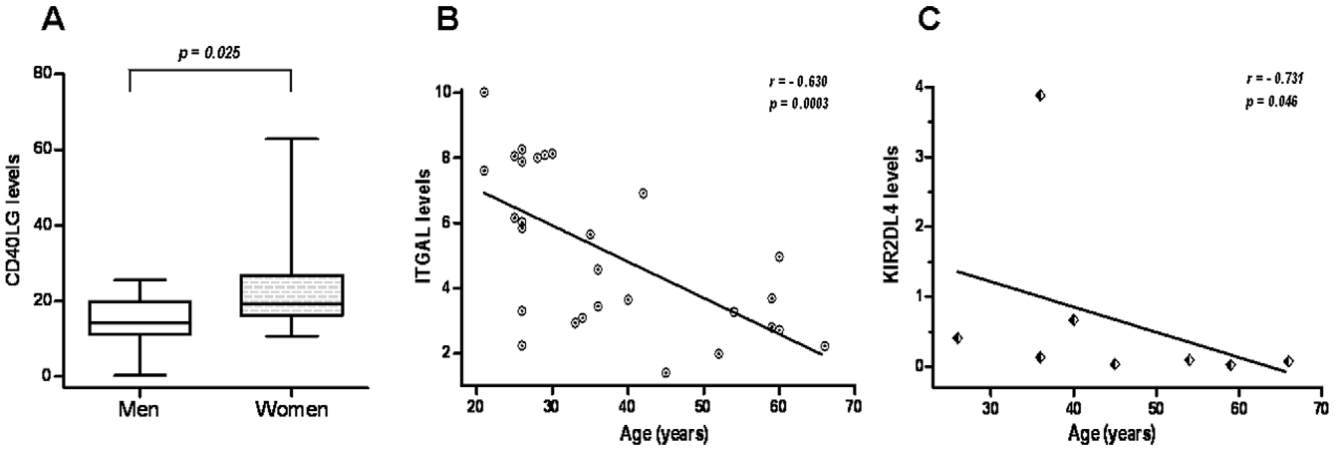
Effect of sex and age on the transcript expression levels of healthy controls' CD4^+^ T cells. A. Women had higher levels of CD40LG than men. Data are presented as box plots, where the lines inside the boxes represent the medians, the boxes represent the 25^th^ and the 75^th^ percentiles, and the lines outside the boxes indicate the highest and lowest value. B, C. Inverse correlations between age and ITGAL (B) and age and KIR2DL4 (C) mRNA levels.

Gender seemed to be no relevant for the expression of ITGAL, PRF1, KIR2DL4, and CD40LG in SLE patients. Just a tendency towards showing a higher expression of CD70 in SLE women was observed (1.65±1.67 vs. 0.68±1.29, p = 0.051). Interestingly, we observed that the 14 women controls included in the study had higher CD40LG mRNA levels than the 16 men controls (23.56±12.93 vs. 14.53±6.93, p = 0.025) (see Figure 1A).

None correlation was observed between age and the expression of the five genes in the SLE population. On the other hand, Spearman's rank negative correlations coefficients were obtained with respect to ITGAL (r = −0.630, p = 0.0003) in the control group (Figure 1B). When we focused on just the 8 healthy controls with KIR2DL4 transcript levels higher than zero, we also found a negative correlation between KIR2DL4 transcript levels and age (r = −0.731, p = 0.046) (Figure 1C).

**Figure 2:**
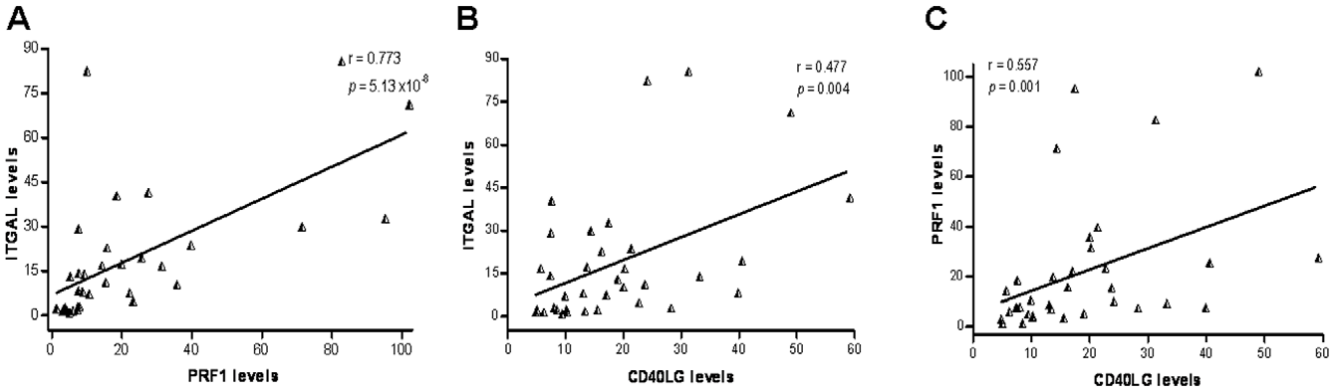
Correlations between transcripts levels of ITGAL and PRF1 (A), ITGAL and CD40LG (B), and PRF1 and CD40LG (C) in CD4+ T cells of patients affected with SLE.

**Figure 3:**
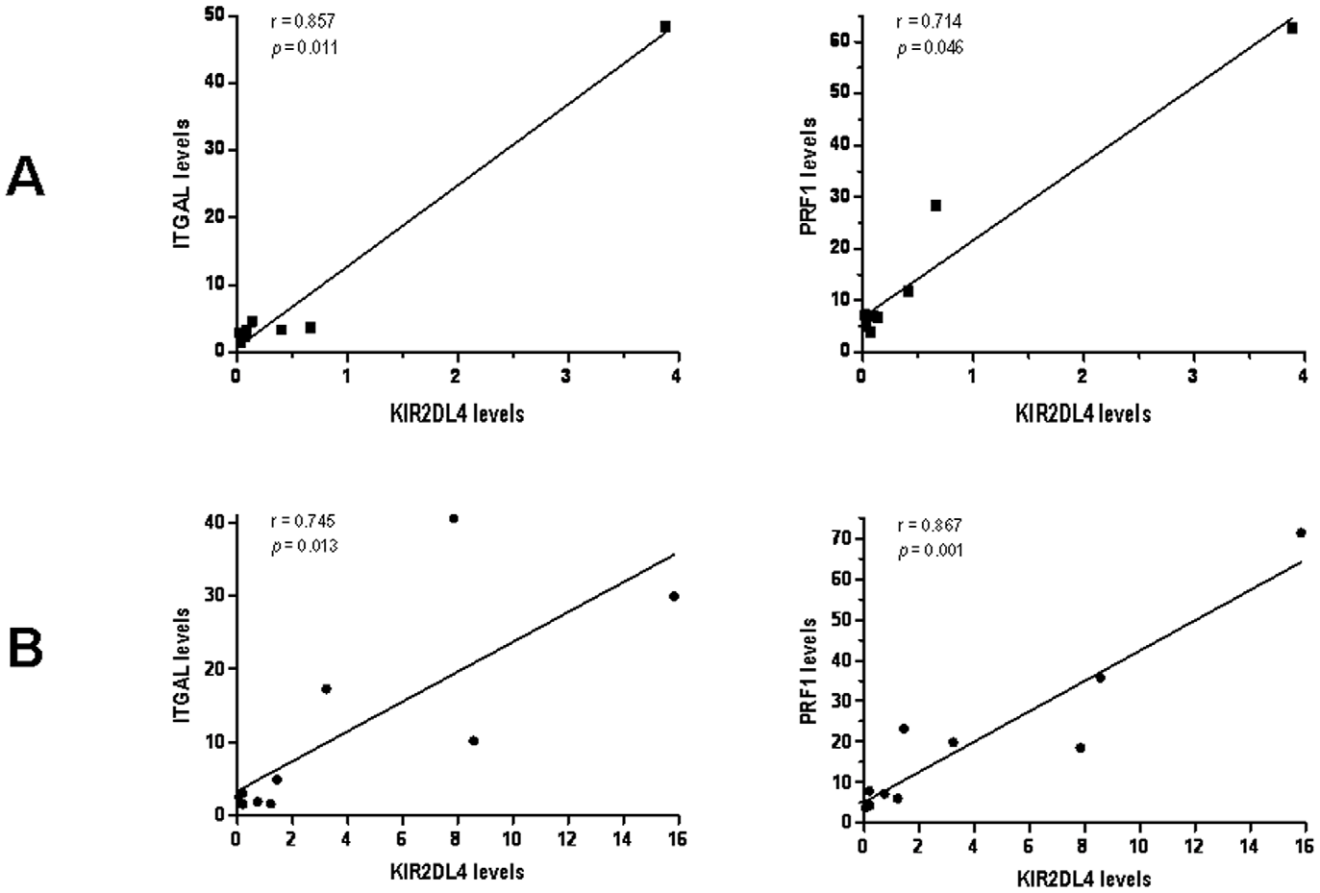
KIR2DL4 transcript levels of CD4^+^ T-cells are proportional to ITGAL and PRF1 mRNA levels both in the healthy control population (A) and the SLE patients group (B).

Relationships between the expression levels of the five genes were also assessed. A strong positive correlation was observed between transcript levels of ITGAL and PRF1 (r = 0.773, p = 5.13×10^−8^), as well as between ITGAL and CD40LG (r = 0.477, p = 0.004) in the patients group. Furthermore, levels of PRF1 and CD40LG seemed to be also positively correlated in SLE individuals (r = 0.557, p = 0.001) (see Figure 2). We then considered those individuals who showed KIR2DL4 transcript levels higher than zero (8 healthy controls and 10 SLE patients). Strong correlations between KIR2DL4 and ITGAL levels were observed in both the control group (r = 0.857, p = 0.011) and the SLE population (r = 0.745, p = 0.013). In the same line, KIR2DL4 levels were also correlated to PRF1 in both populations (r = 0.714, p = 0.046; r = 0.867, p = 0.001, respectively) (Figure 3).

### Global DNA methylation and DNA methylation-related enzymes levels: Effect on the expression of ITGAL, PRF1, KIR2DL4, CD70, and CD40LG

As shown by other authors, in our previous work we also demonstrated that SLE patients had significantly less CD4+ T cell DNA d^m^C content than controls (0.802±0.134 vs. 0.901±0.133; p = 0.007) [8]. Since the expression of ITGAL, PRF1, KIR2DL4, CD70, and CD40LG seems to depend on their promoter's DNA methylation status, we thought it might be interesting to further evaluate the relationship between their transcripts levels and the global DNA d^m^C methylation content of CD4^+^ T cells. In the present study we found that the characteristic low DNA methylation of SLE patients was accompanied by a higher expression of ITGAL (r = −0.443, p = 0.016). As a matter of fact, this inverse correlation with the DNA methylation status was also observed for PRF1 (r = −0.505, p = 0.005), whereas a trend towards this effect was also seen for CD40LG (r = −0.359, p = 0.056) (Table 1).

**Table 1:** Effect of the CD4+ T cell global DNA methylation status and the expression of different DNA methylation-related enzymes (DNMT1, DNMT3A, DNMT3B, MBD2, and MBD4) on the expression of ITGAL, PRF1, KIR2DL4, CD70, and CD40LG.

	*ITGAL*		*PRF*		*KIR2DL4*		*CD70*		CD40LG	
	Controls	Patients	Controls	Patients	Controls	Patients	Controls	Patients	Controls	Patients
***DNA d^m^C content***	**p = 0.028 r = 0.410**	**p = 0.016 r = −0.443**	p = 0.308 r = 0.196	**p = 0.005 r = −0.505**	p = 0.610 r = 0.214	p = 0.397 r = −0.429	p = 0.754 r = 0.061	p = 0.777 r = 0.055	p = 0.172 r = 0.261	**p = 0.056 r = −0.359**
***DNMT1***	**p = 0.022** **r = 0.425**	p = 0.237 r = 0.227	p = 0.743 r = −0.064	p = 0.140 r = 0.281	p = 0.243 r = 0.476	p = 0.497 r = 0.371	p = 0.392 r = 0.165	p = 0.709 r = 0.072	p = 0.218 r = 0.236	p = 0.095 r = 0.316
***DNMT3A***	**p = 3×10^−7^** **r = 0.791**	**p = 0.008 r = 0.483**	p = 0.810 r = 0.047	**p = 0.011 r = 0.467**	p = 0.619 r = 0.214	**p = 0.058** 1 **r = 0.829**	p = 0.810 r = −0.047	p = 0.195 r = 0.248	**p = 0.026 r = 0.412**	**p = 2×10^−4^ r = 0.636**
***DNMT3B***	**p = 2.6×10^−6^ r = 0.781**	**p = 3×10^−4^ r = 0.824**	p = 0.094 r = 0.335	**p = 0.002 r = 0.745**	p = 0.215 r = 0.536	**p = 0.000** 2 **r = 1.000**	p = 0.731 r = −0.071	p = 0.175 r = 0.385	p = 0.120 r = 0.313	**p = 0.034 r = 0.569**
***MBD2***	**p = 0.001** **r = 0.580**	**p = 0.002 r = 0.549**	p = 0.143 r = 0.279	**p = 0.052 r = 0.365**	p = 0.462 r = 0.310	p = 0.356 r = 0.486	p = 0.956 r = 0.011	p = 0.133 r = 0.286	p = 0.449 r = 0.146	**p = 0.027 r = 0.411**
***MBD4***	**p = 6×10^−9^** **r = 0.849**	**p = 8×10^−10^ r = 0.871**	p = 0.115 r = 0.299	**p = 0.002 r = 0.553**	p = 0.151 r = 0.571	**p = 0.058** 1 **r = 0.829**	p = 0.932 r = 0.017	p = 0.187 r = −0.252	p = 0.164 r = 0.266	p = 0.120 r = 0.295

Statistically (or almost statistically) significant correlations are indicated by the p-value and the Spearman's rank correlation coefficient (r) highlighted in bold.

1 Correlation values were established for just the 6 patients from whom paired data were available.

2 Correlation values were established for just the 5 patients from whom paired data were available.

In order to find out the mechanism(s) by which the above associations could be explained, we looked for the possible relationships between the transcript levels of ITGAL, PRF1, KIR2DL4, CD70, and CD40LG and the transcript expression of five DNA-methylation-related enzymes, i.e. three DNA methyltransferases (DNMT1, DNMT3A, and DNMT3B) and two DNA methyl CpG-binding (MBD) proteins (MBD2 and MBD4). In this particular study, paired data were available for 29 controls and 29 SLE patients. As shown in Table 1, levels of ITGAL and PRF1 from SLE patients were directly and strongly correlated with the transcription levels of all the enzymes but DNMT1. When considering those SLE individuals with KIR2DL4 levels higher than zero, we found positive correlations with DNMT3A, DNMT3B, and MBD4. Levels of CD70 were not correlated with the levels of any enzyme. Finally, CD40LG transcript levels were associated to DNMT3A, DNMT3B and MBD2.

As shown in our earlier works, [8], [30] an inverse correlation (or a trend towards it) was seen between global DNA methylation and transcript levels of DNMT1, DNMT3A, DNMT3B, MBD2, and MBD4 in the patients' population (see Table 2). Finally, correlations between the five DNA methylation-related enzymes were also studied. As seen in Table 2, there seemed to be a positive and tight transcription regulation between all of them.

**Table 2:** Correlation study between the CD4+ T cell global DNA methylation status and the expression of different DNA methylation-related enzymes (DNMT1, DNMT3A, DNMT3B, MBD2, and MBD4).

	*DNMT3A*		*DNMT3B*		*MBD2*		*MBD4*		*DNA dm content*	
	Controls	Patients	Controls	Patients	Controls	Patients	Controls	Patients	Controls	Patients
***DNMT1***	**p = 0.024** **r = 0.419**	**p = 1×10^−4^** **r = 0.646**	p = 0.207 r = 0.256	p = 0.220 r = 0.350	p = 0.139 r = 0.281	p = 0.181 r = 0.256	**p = 0.005** **r = 0.503**	p = 0.105 r = 0.308	p = 0.140 r = 0.281	**p = 0.012** **r = −0.459**
***DNMT3A***			**p = 6×10^−6^** **r = 0.763**	p = 0.106 r = 0.451	**p = 0.013** **r = 0.455**	**p = 0.009** **r = 0.474**	**p = 7×10^−10^** **r = 0.872**	**p = 0.011** **r = 0.466**	p = 0.108 r = 0.305	**p = 0.019** **r = −0.432**
***DNMT3B***					**P = 0.005** **r = 0.534**	**p = 0.002** **r = 0.741**	**p = 5×10^−7^** **r = 0.811**	**p = 1×10^−5^** **r = 0.901**	**p = 0.024** **r = 0.443**	**p = 0.078** **r = −0.486**
***MBD2***							**p = 0.001** **r = 0.605**	**p = 0.013** **r = 0.454**	**p = 0.019** **r = 0.433**	**p = 0.008** **r = −0.481**
***MBD4***									**p = 0.025** **r = 0.417**	**p = 0.004** **r = −0.523**

Statistically (or almost statistically) significant correlations are indicated by the p-value and the Spearman's rank correlation coefficient (r) highlighted in bold.

## Discussion

In this work we have determined the transcript expression of five genes that have proved relevant to lupus and we have confirmed previous observations of high expression of three of them: ITGAL, PRF1, and CD70. Overexpression of ITGAL contributes to autorreactivity, [17] whereas abnormal expression of PRF1 contributes to the cytotoxic potential of the autorreactive CD4+ T cells [21]. Interestingly, we found a strong positive correlation between levels of ITGAL and PRF1. Of note, Kaplan *et al*. [21] also found that PRF1 was preferentially expressed in T cells with high LFA-1 levels. LFA-1–mediated signaling mechanisms induce the release of perforin in NK cells [33]. We could hypothesize that a high expression of ITGAL may usually activate not only the release but also the transcription of PRF1 in CD4+ T cells. On the other hand, the simultaneous high transcription of ITGAL and PRF1 detected in CD4^+^ T cells from SLE patients indicates that both proteins may act synergetically. ITGAL could help to overstabilize the normally low affinity interaction between the T-cell receptor (TCR) and the Major Histocompatibility Complex MHC class II molecules (thus decreasing the antigen threshold dose on the presenting cell for T cell activation) and PRF1 would exert its cytolitic activity. It all would promote the overt apoptosis observed in these patients [34].

Regarding gender, we observed a tendency towards showing a higher expression of CD70 in SLE women. Oelke *et al*. and Kozlowska *et al*. [23], [35] included basically only SLE women in their studies (13 out of 14, and 39 out of 41, respectively) and they also found that CD70 was overexpressed. Since in our work the mean levels of CD70 for male SLE were similar to the ones detected in controls, we can not rule out the possibility that the overexpression of CD70 may indeed be found just in female patients.

Lu *et al*. [24] have reported that CD4^+^ T cells from women but not men with lupus overexpress CD40LG and it seems to be due to the demethylation of the inactive X chromosome in females [25]. We did not find such overexpression neither when comparing genders nor when comparing the whole SLE population with the control group. On the contrary, our healthy women did present higher CD40LG levels than our healthy men. Besides the fact that Lu *et al*. [24] only analysed CD40LG mRNA levels from three individuals in each group, other reasons may account for this discrepancy. As opposed to these studies, we did not stimulate the cells. Healthy and patients' cells could behave differently under culture conditions. Since we worked directly with non-manipulated lymphocytes, we think that our findings may reflect in a more accurate way the real *in vivo* expression of CD40LG. Nevertheless, it was quite remarkable to see that healthy women had higher transcript levels of CD40LG than healthy men. It could indicate that in normal situations CD40LG does escape X-chromosome inactivation. Evidently, further studies are needed to confirm such finding.

In general, absolute numbers of T cells that express surface CD40LG are increased in patients with SLE [36]. Therefore, our results were somehow unexpected. In previous studies we have shown that SLE patients have significantly lower levels of soluble CD40LG (sCD40LG) during flare than during remission [37]. The same statement might hold true for transcript levels of CD40LG in CD4^+^ T cells. Actually, levels of CD40LG transcripts in SLE blood lymphocytes correlate with the relative concentrations of sCD40LG found in SLE plasma [38]. Since in the present work all but four of our patients where at flare, we may conclude that the fact we did not find an increase on CD40LG levels in our SLE patients was because most of them were very active from a clinical point of view. In order to clarify this issue, we encourage other authors to further evaluate the expression of CD40LG taking into account the illness status of the patient. The fact that we found positive correlations of CD40LG levels with the levels of two overexpressed molecules (ITGAL and PRF1), leads us to infer that CD40LG probably would also be overexpressed if we had considered a more diverse group of SLE patients at different stages of the disease.

Basu *et al*. [22] reported high KIR protein levels on lupus T cells. In their study they used a cocktail of antibodies directed against different KIRs and although they evaluated the methylation status of the KIR2DL4 promoter, they did not show whether the observed hypomethylation correlated with high levels of transcripts. We did not find an overexpression of KIR2DL4 mRNA in our patients. Unlike ITGAL, PRF1, CD70, and CD40LG, it is possible that KIR2DL4 do not represent a marker of the disease. In fact, few patients showed any expression at all and, although positive correlations were found between KIR2DL4 levels and ITGAL and PRF1 levels, we must keep in mind that they were also found in the controls.

T-cell DNA of SLE patients is globally hypomethylated [7]–[9]. The degree of DNA methylation has also been analysed at the promoter level on ITGAL [19], PRF1, [21] KIR2DL4, [22] CD70, [39] and CD40LG, [24] and it has been shown to be low in SLE. In this work we wanted to ascertain whether the global DNA hypomethylation seen in our patients was linked to a higher transcription of the five genes under study. Both ITGAL and PRF1 transcript levels increased as global DNA methylation decreased and a trend was also observed for CD40LG. Thus, although we did not specifically analyse their promoters, global DNA methylation gives us an indication that ITGAL, PRF1 and CD40LG really are epigenetically-regulated genes. A negative correlation between DNA global methylation levels and ITGAL was also described by Luo *et al.* [15] in CD4^+^ T cells from subacute cutaneous lupus patients.

CD70 and KIR2DL4 levels did not show any correlation with global DNA methylation. Hence, although their promoters are known to be methylation-sensitive, global DNA methylation seems to be a non-sufficiently strong marker to unveil their true nature.

The DNA methylation effects described above could ultimately be attributed to the expression of specific DNA methylation-related enzymes. ITGAL levels correlated pretty well with DNMTs and MBDs levels. Similar positive correlations were observed for PRF1 and CD40LG in the patients population. Although some associations were found for KIR2DL4, very few individuals were included in these analyses and, consequently, reliable conclusions can not be withdrawn. CD70 expression has been proved to be enhanced by DNA methylation inhibitors; [23] nonetheless, we did not find any indication that DNMTs or MBDs may be linked to its regulation. Therefore, we believe that it is possible that other mechanisms (which would not directly involve the five DNA methylation enzymes included in our study) participate in its regulation. As demonstrated by other authors, both aberrant histone modifications within the CD70 promoter, as well as certain regulatory factors may contribute to the increase of CD70 expression in SLE [40], [41].

What seems to be clear is that ITGAL, PRF1, and CD40LG levels were simultaneously and directly linked to the expression of DNMTs and MBDs and inversely correlated to global DNA methylation. Wei-Min et al. [42] also found that transcript levels of ITGAL correlated with those of MBD2. In our previous works [8], [30] we reported high levels of MBD2 and MBD4 in SLE CD4^+^ T cells. Other authors have corroborated such findings [14], [15], [42]. On the other hand, we did not find the decreased levels of DNMTs observed by other groups [10], [11]. Instead, we realized that mRNA levels of DNMTs increased in response to the underlying DNA hypomethylation status when the disease was being definitely active. In summary, our findings led us to conclude that MBD2 and MBD4 seemed to have a direct and active role by demethylating CD4^+^ T cell DNA of SLE patients whereas DNMTs increased accordingly in an attempt to overcome hypomethylation. As seen in the present work, a tight and clear-cut transcription regulation among the three DNMTs and the two MBDs exists. As a consequence, and based in our results, we could establish the following hypothesis: overexpression of MBD2 and MBD4 may cause DNA hypomethylation which, in turn, would activate some genes involved in the immune response; although not overexpressed, DNMTs would increase their levels to try to methylate (i.e., silence) those genes whose overexpression would be detrimental to the subject's health. We could approach the idea, though, from another completely different perspective. Thus, one could think that the increase on MBD2 and MBD4 would emerge in an attempt to silence these genes overexpressed in SLE. To consider this second hypothesis we have to accept that, as described by other authors, MBDs can function as transcriptional repressors [43].

In summary, our investigation demonstrates that the expression of ITGAL, PRF1, and CD70 is upregulated in CD4^+^ T cells from SLE patients. Although CD40LG transcript levels were not found to be elevated in our SLE patients, its tight association with ITGAL and PRF1 leads us to infer that it probably contributes to the pathogenesis of the disease. On the other hand, based on our results, the significance of KIR2DL4 would be controversial. The relationships established between their expression and the global DNA methylation content as well as with the transcription of different DNA methylation-related enzymes, indicates that epigenetic mechanisms are responsible for the deregulation of ITGAL, PRF1, and CD40LG. Thus, our work reinforces the importance of abnormal DNA methylation in the development of SLE and provides insights into the molecular bases that underlie the expression of these autoimmune-related genes.
